# miR-31-5p from placental and peripheral blood exosomes is a potential biomarker to diagnose preeclampsia

**DOI:** 10.1186/s41065-022-00250-z

**Published:** 2022-09-19

**Authors:** Gang Zou, Qingfang Ji, Zixiang Geng, Xiling Du, Lingyan Jiang, Te Liu

**Affiliations:** 1 Shanghai Key Laboratory of Maternal Fetal Medicine, Department of Fetal Medicine & Prenatal Diagnosis Center, Shanghai First Maternity and Infant Hospital, School of Medicine, Tongji University, Shanghai, 200092 China; 2 Shuguang Hospital, Shanghai University of Traditional Chinese Medicine, Shanghai, 201203 China; 3 School of Life Science and Technology, Tongji University, Shanghai, 200092 China; 4 Department of Gynecology and Obstetrics, Shanghai Fourth People’s Hospital, School of Medicine, Tongji University, 1279, Sanmen Road, Shanghai, 200434 China; 5 Shanghai Geriatric Institute of Chinese Medicine, Shanghai University of Traditional Chinese Medicine, 365 South Xiangyang Road, Shanghai, 200031 China

**Keywords:** Preeclampsia, Exosome, microRNA, Biomarker

## Abstract

**Background:**

Preeclampsia, a multisystem disorder of unknown etiology, is one of the leading causes of maternal and perinatal morbidity and mortality. Identifying sensitive, noninvasive markers can aid its prevention and improve prognosis. microRNAs (miRs), which function as negative regulators of gene expression, are closely related to preeclampsia occurrence and development. Herein we investigated the relationship between the *DLK1-Dio3* imprinted miR cluster derived from placental and peripheral blood exosomes of pregnant women with preeclampsia and routine clinical diagnostic indicators, and also determined its potential as a noninvasive diagnostic marker.

**Methods:**

Exosomes were extracted from the placenta and peripheral blood of pregnant women with preeclampsia.

**Results:**

qPCR data indicated that the expression level of miRs, such as miR-134, miR-31-5p, miR-655, miR-412, miR-539, miR-409, and miR-496, in pregnant women with preeclampsia was significantly lower than that in healthy controls; miR-31-5p expression was the most different. Gene ontology analysis predicted that genes negatively regulated by miR-31-5p were mainly enriched in cellular entity, cellular process, and binding; moreover, Kyoto Encyclopedia of Genes and Genomes pathway analyses indicated that genes were involved in gonadotropin-releasing hormone receptor pathway and other signaling pathways. Correlation analysis revealed that miR-31-5p was significantly negatively correlated with clinical indicators of preeclampsia, such as systolic and diastolic pressure, lactate dehydrogenase, and proteinuria.

**Conclusion:**

We believe that exosome-derived miR-31-5p can serve as an effective and sensitive biomarker to determine the course of preeclampsia in pregnant women.

## Introduction

Preeclampsia is associated with high morbidity and mortality. It affects 3–5% of all pregnancies in the US and leads to > 500,000 preterm births and 70,000 maternal deaths worldwide each year [1–4]. New-onset hypertension, characterized by proteinuria and end-organ dysfunction, is the most typical clinical manifestation of preeclampsia and usually occurs after 20-week gestation [1–4]. Preeclampsia can be broadly classified into two categories: placental and maternal. The former stems from a diseased placenta [1–4]. The only known cure for preeclampsia is delivery of the placenta, which can potentially resolve most signs and symptoms; however, this approach commonly correlates with iatrogenic preterm delivery [1–4]. Therefore, further studying the pathogenesis of preeclampsia and identifying a sensitive biomarker should contribute to its prevention, early intervention, and treatment.

microRNAs (miRs) are endogenous single-stranded non-coding RNAs with no open reading frames; they range from 19 nt to 25 nt in length and are primarily produced by the ribonuclease III enzymes Drosha and Dicer [5, 6]. miRs elicit their regulatory effects by binding to the 3′-untranslated region of target mRNAs; this induces specific cleavage of the target mRNA by Dicer, resulting in mRNA degradation and translational inhibition of functional proteins [7–9]. In animals, miRs are involved in almost all physiological development and pathological processes, such as cell proliferation, differentiation, migration, and apoptosis [5, 6]. The *DLK1-Dio3* imprinted miR cluster has attracted considerable attention. The approximately 1-Mbp eutherian-specific imprinted *Dlk1-Dio3* chromosomal region is located on distal mouse chromosome 12 (orthologous to human chromosome 14q32). In humans, this chromosomal region comprises three paternally expressed protein-coding genes (i.e., *Dlk1*, *Rtl1*, and *Dio3*) and numerous maternally expressed non-protein-coding genes, including MEG3 (Gtl2), MEG8 (RIAN), and antisense RTL1. miRs in this region are prominently expressed in embryos, adult tissues and organs, and placental tissues [8, 10–12]; moreover, they have important regulatory roles in the maintenance of mammalian stem cell pluripotency, embryonic development, organogenesis, and tumorigenesis [13–17]. Firstly, Takeshi Saito et al. found that a tandem repeat array in IG-DMR was essential for imprinting of paternal allele at the *DLK1-Dio3* domain during embryonic development [18]. And, Maria Schacker et al. reported that hypermethylation and down-expression of Gtl2, Rian and Mirg at the *DLK1-Dio3* imprinted locus were positive correlations of poor developmental potential of mouse embryonic stem cells [19]. Chu-Fan Mo et al. indicated that loss of non-coding RNA expression from the *DLK1-Dio3* imprinted locus correlates with reduced neural differentiation potential in human embryonic stem cell lines [20]. Thus, many studies have pointed out that the *DLK1-Dio3* imprinted clusters have important regulatory roles in the maintenance of embryonic development. However, the role of the *DLK1-Dio3* imprinted miR cluster in preeclampsia occurrence and development has not been reported.

Exosomes are saucer-shaped extracellular vesicles with a diameter of 50–100 nm and density of 1.13–1.19 g/mL. They are surrounded by a lipid bilayer membrane, and they are released into the extracellular environment upon the fusion of multivesicular bodies with the plasma membrane [21–24]. An increasing number of studies have found that exosomes can deliver membrane and cytoplasmic proteins from one cell to another and that they function as carriers to deliver miRs into cells. Furthermore, exosomes act as messengers of cellular transformation and participate in diverse physiological and pathophysiological processes [21–24]. Exosomes can package a large number of miRs and effectively transport them to recipient cells; in these cells, exogenous miRs silence specific target genes and trigger downstream signaling events, further affecting the function and state of target cells [25–27]. Although the mechanism of miR release via exosome encapsulation to the extracellular environment remains unclear, studies have suggested that miR encapsulation into microvesicles is an active process; some specific miR populations are apparently selectively encapsulated into microvesicles under certain pathological or physiological conditions [25–27]. Li et al. indicated that exosomes not only serve as miR carriers but also protect miRs from degradation [28]. In addition, exosomes secreted by cells contain not only functional components of RNA-induced silencing complex to enhance miR function but also argonaute-2 protein complexes to protect miR degradation by RNA enzymes [28]. We previously reported that the contents of peripheral blood exosomes (e.g., circRNA, miR, and hormones) are potential markers for the prognosis of infertility and hepatocellular carcinoma [7, 29, 30]. Whether miRs in maternal peripheral blood (and/or placental) exosomes are closely related to preeclampsia remains to be reported.

In this study, we assessed pregnant women with preeclampsia and healthy pregnant women to evaluate differences in expression levels of the *DLK1-Dio3* imprinted miR cluster. Our aim was to elucidate the consistency in miR expression levels between peripheral blood exosomes and placental exosomes, as well as to determine the relationship with routine clinical diagnostic indicators of preeclampsia. Our findings provide an experimental basis for establishing peripheral blood exosome–miRs as a marker of preeclampsia in pregnant women.

## Materials and methods

All human materials were obtained according to consent regulation and approved by the Ethics Review Committee of Shanghai Geriatric Institute of Chinese Medicine of Research in Human Production (authorized by Shanghai Municipal Government). All patients provided informed consent in accordance with the Declaration of Helsinki.

### Exosome isolation from placental tissues

Placental tissues were collected from pregnant women with preeclampsia at Shanghai First Maternal and Infant Health Care Hospital from January 2020 to December 2021. As previously reported [7, 29–31], briefly, the tissue was digested with 0.25% trypsin (containing 0.05% EDTA); cell suspension was collected and centrifuged at 500×*g* for 10 min for the removal of cell fragments and apoptotic bodies. The supernatant was then centrifuged [SORVALL® Discovery™ SE Ultracentrifuge with a SORVALL® T-865 Fixed 23.5° Angle Rotor, k-factor = 51.7 (Thermo Scientific)] at 16,500×*g* for 20 min to collect exosomes. The supernatant was passed through a 0.45-μm filter to remove protein and debris. Subsequently, exosomes were precipitated by centrifugation at 118,000×*g* for 70 min.

### Exosome isolation from peripheral blood

Peripheral blood samples were collected from pregnant women with preeclampsia at Shanghai First Maternal and Infant Health Care Hospital from January 2020 to December 2021. As previously reported [7, 29–31], briefly, peripheral blood was centrifuged at 500×*g* for 10 min for serum collection, and serum samples were then centrifuged [SORVALL® Discovery™ SE Ultracentrifuge with a SORVALL® T-865 Fixed 23.5° Angle Rotor, k-factor = 51.7 (Thermo Scientific)] at 16,500×*g* for 20 min for exosome collection. The supernatant was passed through a 0.45-μm filter to remove protein and debris. Finally, exosomes were precipitated by centrifugation at 118,000×*g* for 70 min.

### RNA extraction and reverse-transcription

As per the manufacturer instructions of RNAprep Pure Tissue Kit [TIANGEN Biotech (Beijing) Co., Ltd], approximately 20 mg human tissue sample and 800 μL lysate were mixed, and the homogenate was ground. To the supernatant, 200 μL chloroform was added, followed by thorough mixing and centrifugation at 13,400×*g* and 4 °C for 15 min. To the supernatant thus obtained, anhydrous ethanol (two times the volume of the supernatant) was added; this suspension was thoroughly mixed and centrifuged at 13,400×*g* and 4 °C for 30 min. RNA precipitate was then suspended in 500 μL 75% ethanol, followed by centrifugation at 13,400×*g* and 4 °C for 5 min. Any excess liquid was removed, and 300 μL diethylpyrocarbonate-treated water was added to the precipitate. Subsequently, to determine RNA purity and total concentration, 1 μL RNA solution was used to calculate OD_260_/OD_280_ (generally between 1.8 and 2.0). According to the instructions of miRcute miRNA first-strand cDNA Synthesis kit [TIANGEN Biotech (Beijing) Co., Ltd], 20 μL total RNA (100 ng/μL) was thoroughly mixed with 25 μL 2× miRNA RT Reaction Buffer, 4 μL 1× miRNA RT Enzyme Mix, and 6 μL RNase-free deionized water. The following reactions were carried out in the PCR apparatus, and miR plus A tail and reverse transcription reactions were performed at 42 °C for 60 min. The enzyme inactivation reaction was performed at 95 °C for 3 min.

### Quantitative real-time PCR (qPCR)

As per the manufacturer instructions of miRcute miRNA qPCR detection kit [TIANGEN Biotech (Beijing) Co., Ltd], reagents, test samples, and primers were mixed: 10 μL 2× miRNA Premix (with SYBR), 1 μL 1× forward and reverse primers (10 μM), 4 μL first-strand cDNA of microRNA, and 4 μL deionized water. For qPCR, the cycling conditions were as follows: incubation at 95 °C for 15 min, followed by 40 cycles of 94 °C for 20 s and 60 °C for 34 s. Fluorescence values were recorded. The primers are as follows: hsa-mir-1185-1-F: 5′-atatacagggggagactcttat-3′; hsa-mir-1185-2-F: 5′-atatacagggggagactctcat-3′; hsa-mir-1197-F: 5′-taggacacatggtctacttct-3′; hsa-mir-1247-F: 5′-ccccgggaacgtcgagactggagc-3′; hsa-mir-127-F: 5′-tcggatccgtctgagcttggct-3′; hsa-mir-134-F: 5′-cctgtgggccacctagtcaccaa-3′; hsa-mir-136-F: 5′-actccatttgttttgatgatgga-3′; hsa-mir-154-F: 5′-taggttatccgtgttgccttcg-3′; hsa-mir-299-F: 5′-tatgtgggatggtaaaccgctt-3′; hsa-mir-300-F: 5′-tatacaagggcagactctctct-3′; hsa-mir-323-F: 5′-acacattacacggtcgacctct-3′; hsa-mir-323b-F: 5′-cccaatacacggtcgacctctt-3′; hsa-mir-329-F: 5′-aacacacctggttaacctcttt-3′; hsa-mir-369-F: 5′-aataatacatggttgatcttt-3′; hsa-mir-370-F: 5′-gcctgctggggtggaacctggt-3′; hsa-mir-376a-F: 5′-atcatagaggaaaatccacgt-3′; hsa-mir-376b-F: 5′-atcatagaggaaaatccatgtt-3′; hsa-mir-376c-F: 5′-aacatagaggaaattccacgt-3′; hsa-mir-377-F: 5′-atcacacaaaggcaacttttgt-3′; hsa-mir-379-F: 5′-tggtagactatggaacgtagg − 3′; hsa-mir-380-F: 5′-tatgtaatatggtccacatctt-3′; hsa-mir-381-F: 5′-tatacaagggcaagctctctgt-3′; hsa-mir-382-F: 5′-aatcattcacggacaacactt-3′; hsa-mir-409-F: 5′-gaatgttgctcggtgaacccct-3′; hsa-mir-410-F: 5′-aatataacacagatggcctgt-3′; hsa-mir-411-F: 5′-tagtagaccgtatagcgtacg-3′; hsa-mir-412-F: 5′-acttcacctggtccactagccgt-3′; hsa-mir-421-F: 5′-atcaacagacattaattgggcgc-3′; hsa-mir-431-F: 5′-tgtcttgcaggccgtcatgca-3′; hsa-mir-432-F: 5′-tcttggagtaggtcattgggtgg-3′; hsa-mir-433-F: 5′-atcatgatgggctcctcggtgt-3′; hsa-mir-487a-F: 5′-aatcatacagggacatccagtt-3′; hsa-mir-487b-F: 5′-aatcgtacagggtcatccactt-3′; hsa-mir-494-F: 5′-tgaaacatacacgggaaacctc-3′; hsa-mir-495-F: 5′-aaacaaacatggtgcacttctt-3′; hsa-mir-496-F: 5′-tgagtattacatggccaatctc-3′; hsa-mir-539-F: 5′-atcatacaaggacaatttcttt-3′; hsa-mir-541-F: 5′-tggtgggcacagaatctggact-3′; hsa-mir-543-F: 5′-aaacattcgcggtgcacttctt-3′; hsa-mir-544a-F: 5′-attctgcatttttagcaagttc-3′; hsa-mir-654-F: 5′-tatgtctgctgaccatcacctt-3′; hsa-mir-655-F: 5′-ataatacatggttaacctcttt-3′; hsa-mir-656-F: 5′-aatattatacagtcaacctct-3′; hsa-mir-668-F: 5′-tgtcactcggctcggcccactac-3′; hsa-mir-758-F: 5′-tttgtgacctggtccactaacc-3′; hsa-mir-770-F: 5′-tccagtaccacgtgtcagggcca-3′; hsa-mir-889-F: 5′-ttaatatcggacaaccattgt-3′; h18s rRNA-F-F: 5′-cagccacccgagattgagca-3′; h18s rRNA-R-F: 5′-tagtagcgacgggcggtgtg-3′.

### Hematoxylin–eosin (H&E) staining

Tissue samples were fixed in 4% paraformaldehyde, dehydrated, and paraffin embedded. A 4-μm paraffin-embedded section was then obtained, which was dewaxed in xylene and dehydrated in an ethanol gradient. Staining was performed with hematoxylin at room temperature for 5 min, followed by differentiation with 1% hydrochloric acid ethanol for 30 s. Light ammonia water was added to turn the section blue for 1 min, and the section was then rinsed for 5 min with distilled water. Subsequently, staining was performed with eosin at room temperature for 2 min, followed by rinsing with distilled water for 2 min. Ethanol gradient decolorization was performed. Xylene was allowed to permeate for 2 min. Finally, the sample was sealed with neutral gum.

### Transmission electron microscopy (TEM)

Exosome samples were coated on a copper mesh and stained with 1% uranium dioxyacetate (Sigma-Aldrich, St. Louis, USA) and 1% lead citrate (Sigma-Aldrich). The samples were then observed under a JEM-1230 transmission electron microscope (JEOL, Japan) and images were captured.

### Statistical analysis

Each experiment was performed at least three times. Values represent mean ± standard error. Data were analyzed using Student’s *t*-test, when appropriate. Differences were considered significant at *P* < 0.05.

## Results

### Expression of the DLK1-Dio3 imprinted miR cluster in placental exosomes

**Fig. 1:**
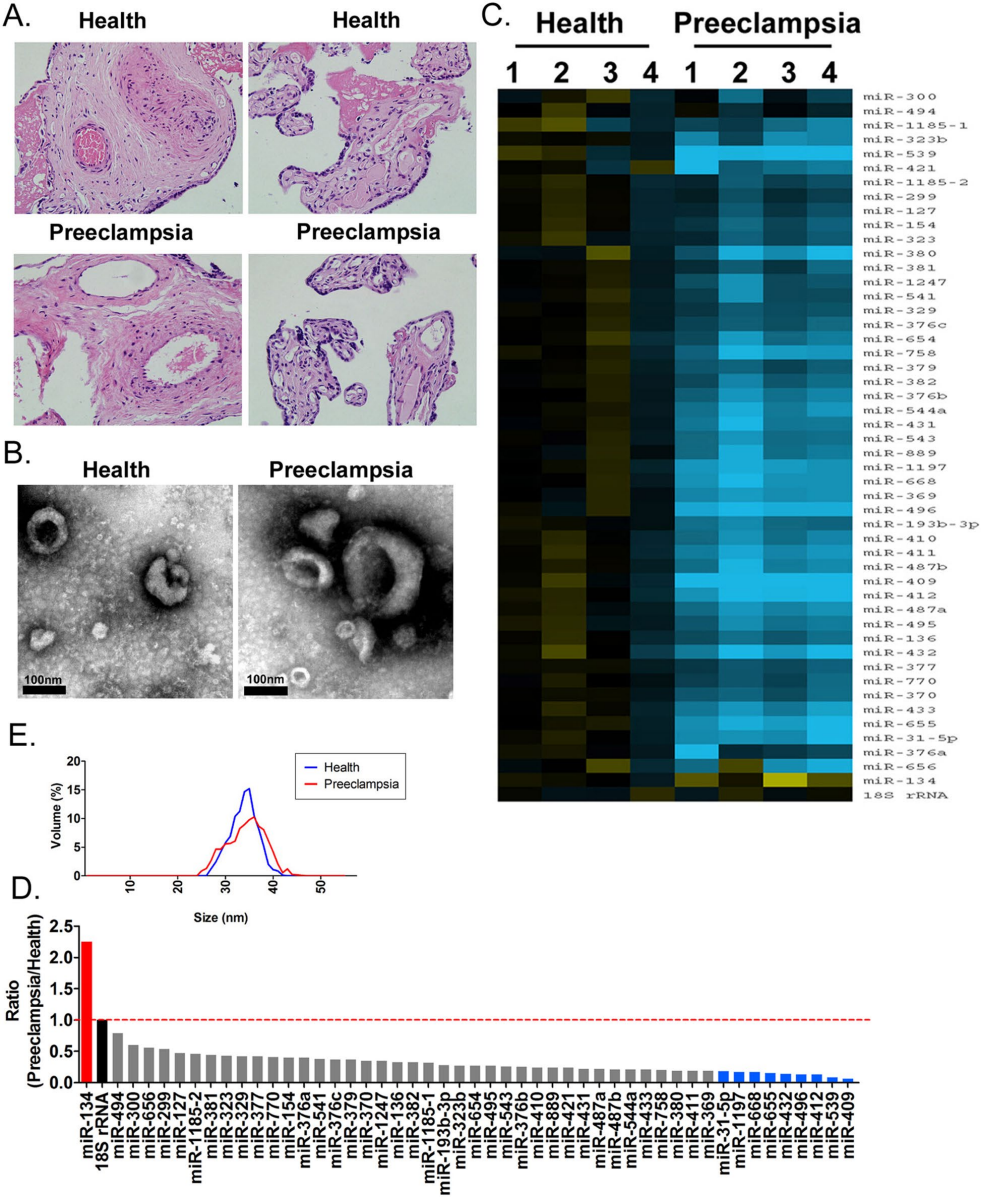
Differential expression of the *DLK1-Dio3* imprinted miR cluster in placental exosomes of pregnant women with preeclampsia. **A** H&E staining of placental tissues. Magnification, 400×. **B** TEM of exosomes. Magnification, 1500×; scale bar, 100 nm. **C** Particle size detection. **D** Heatmap showing expression levels of the *DLK1-Dio3* imprinted miR cluster. **E** qPCR results

We first examined placental tissues of pregnant women with preeclampsia (*n* = 4) and healthy pregnant women (*n* = 4). In case of pregnant women with preeclampsia, pathological examination of placental tissues revealed proliferation of trophoblasts, increased syncytial nodules, cellulose deposition around villi, obvious villous fibrinoid necrosis, edema of placental blood vessels and narrowing of lumen, increased villous blood vessels, and thickening of the vascular basement membrane. Extensive villi hemorrhage and placental infarcts were evident. In contrast, in case of healthy pregnant women, placental villi showed uniform stroma and intact vascular structure, with occasional hemorrhagic spots and villous fibroid necrosis (Fig. 1A-B). TEM demonstrated that both groups secreted abundant exosomes; further, the diameter of placental exosomes in case of both groups was 20–50 nm (Fig. 1E). These findings were consistent with those previously reported.

To analyze expression levels of the *DLK1-Dio3* imprinted miR cluster, qPCR was performed using placental exosomes from pregnant women with preeclampsia and healthy pregnant women. We found 49 miRs in the *DLK1-Dio3* imprinted miR cluster (Fig. 1C). The expression level of 10 miRs (miR-134, miR-31-5p, miR-1197, miR-668, miR-655, miR-432, miR-496, miR-412, miR-539, and miR-409) was significantly different between the groups (Fig. 1D). miR-134 expression in placental exosomes obtained from pregnant women with preeclampsia was significantly upregulated; expression levels of all other miRs were significantly downregulated.

### Expression of the DLK1-Dio3 imprinted miR cluster in placental and peripheral blood exosomes

**Fig. 2:**
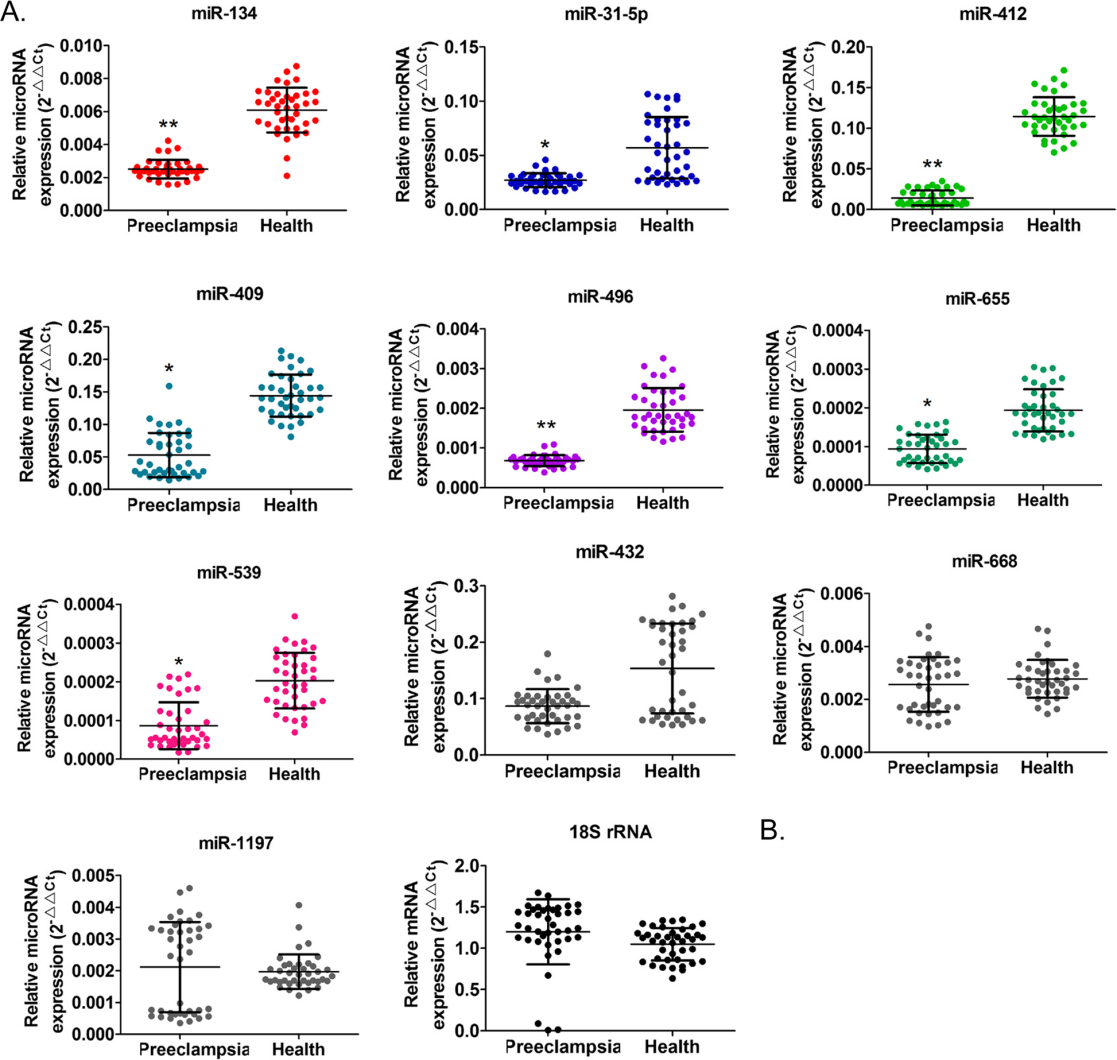
Differential expression levels of the *DLK1-Dio3* imprinted miR cluster in placental exosomes.**P < 0.01 vs healthy controls; *P < 0.05 vs healthy controls; *n* = 40; *t*-test

To verify the accuracy of the aforementioned results, we collected placental tissues from 40 pregnant women with preeclampsia and 40 healthy women, followed by exosome isolation. qPCR was performed to validate expression levels of the following 10 miRs: miR-134, miR-31-5p, miR-1197, miR-668, miR-655, miR-432, miR-496, miR-412, miR-539, and miR-409. miR-1197, miR-668, and miR-432 expression levels did not show significant statistical differences in the identification of expanded samples (Fig. 2). miR-134 expression in placental exosomes showed significant differences between the groups (*P*<0.01); however, this result was contrary to the above results (*n* = 4). The expression levels of the remaining six miRs in placental exosomes obtained from pregnant women with preeclampsia were significantly lower than those in healthy pregnant women (Fig. 2).

**Fig. 3:**
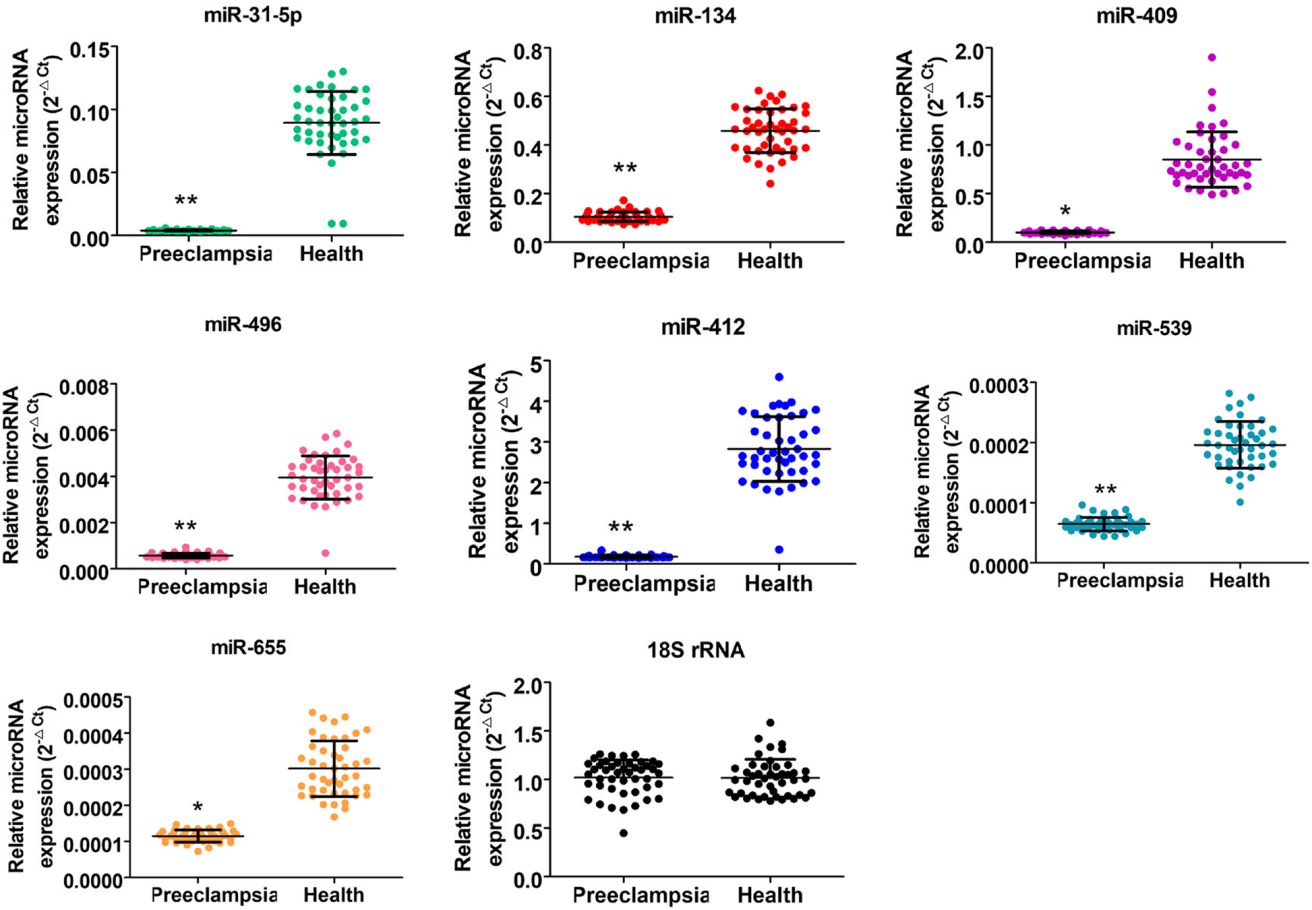
Differential expression levels of the *DLK1-Dio3* imprinted miR cluster in peripheral blood exosomes. **P < 0.01 vs healthy controls; *P < 0.05 vs healthy controls; *n* = 40; *t*-test

Further, we assessed exosomes from peripheral blood samples. qPCR results indicated that the expression trend of miRs, such as miR-134, miR-31-5p, miR-655, miR-412, miR-539, miR-409, and miR-496, was consistent between peripheral blood and placental exosomes (Fig. 3); i.e., the expression level of exosomes in peripheral blood of pregnant women with preeclampsia was significantly lower than that in healthy pregnant women. miR-31-5p expression showed the largest difference between the groups (*P* < 0.01) (Fig. 3). These results revealed that the *DLK1-Dio3* imprinted miR cluster showed similar expression trend in placental and peripheral blood exosomes obtained from pregnant women with preeclampsia.

### miR-31-5p from placental and peripheral blood exosomes

**Fig. 4:**
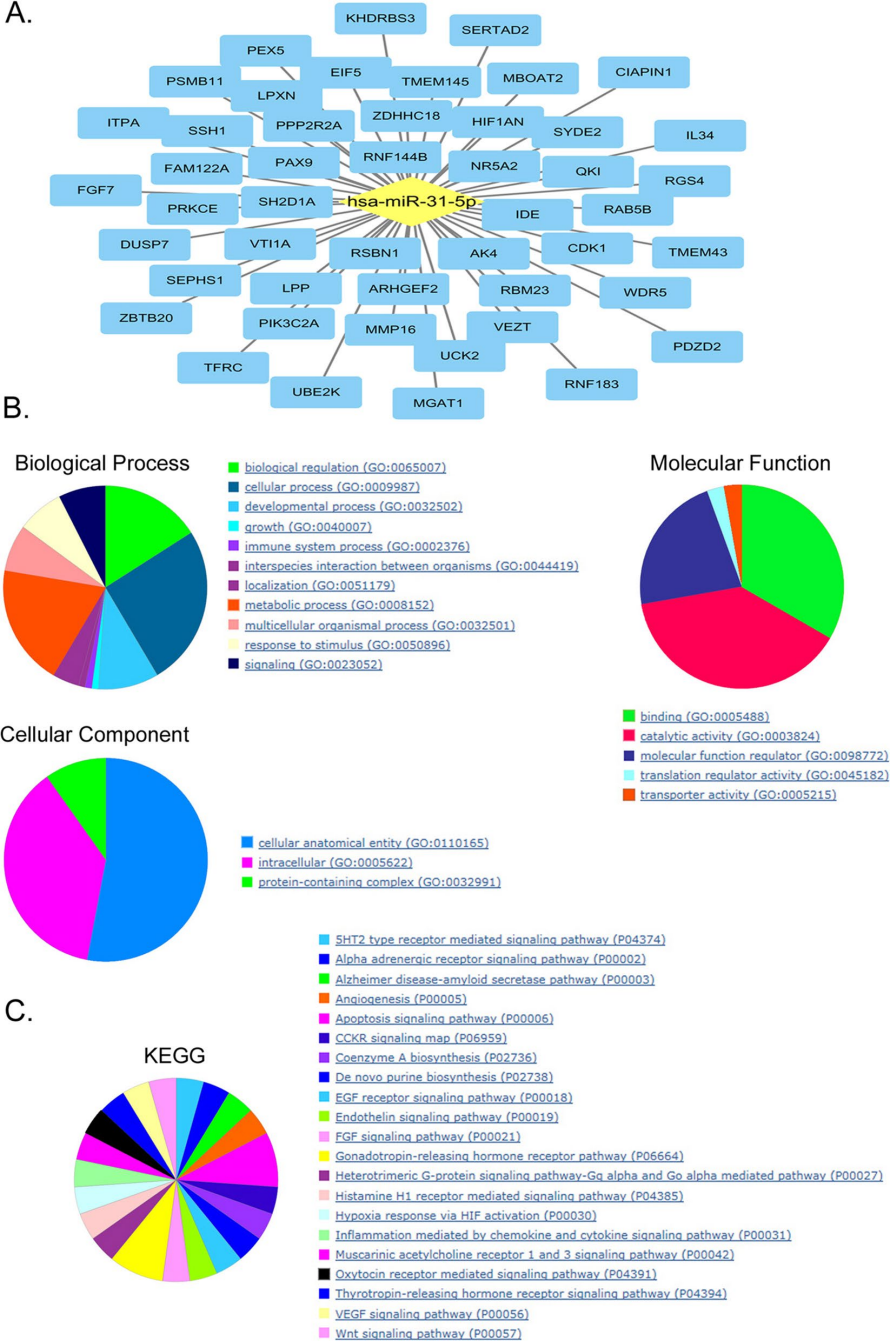
miR-31-5p from placental and peripheral blood exosomes in pregnant women with preeclampsia targets multiple genes. **A** Prediction of negative regulatory target genes. **B**, **C** Function prediction of target genes by gene ontology and Kyoto Encyclopedia of Genes and Genomes pathway analyses

Using TargetScan v8.0 (https://www.targetscan.org), miR-31-5p was found to negatively regulate several types of genes, such as those encoding the cytokines FGF7 and IL34, transcription factor HIF1AN, cell cycle regulatory factor CDK1, and RING finger protein RNF183 (Fig. 4A). PANTHER v17.0 (http://www.pantherdb.org) was used to systematically analyze and predict the function of candidate target genes. Gene ontology analysis revealed that genes negatively regulated by miR-31-5p were mainly enriched in binding (molecular function), cellular entity (cellular component), and cellular process (biological process) (Fig. 4B). Besides, Kyoto Encyclopedia of Genes and Genomes (KEGG) pathway analysis indicated that target genes were mainly involved in gonadotropin-releasing hormone receptor pathway (Fig. 4C).

### Negative correlation between exosome-derived miR-31-5p and routine clinical diagnostic indicators of preeclampsia

**Fig. 5:**
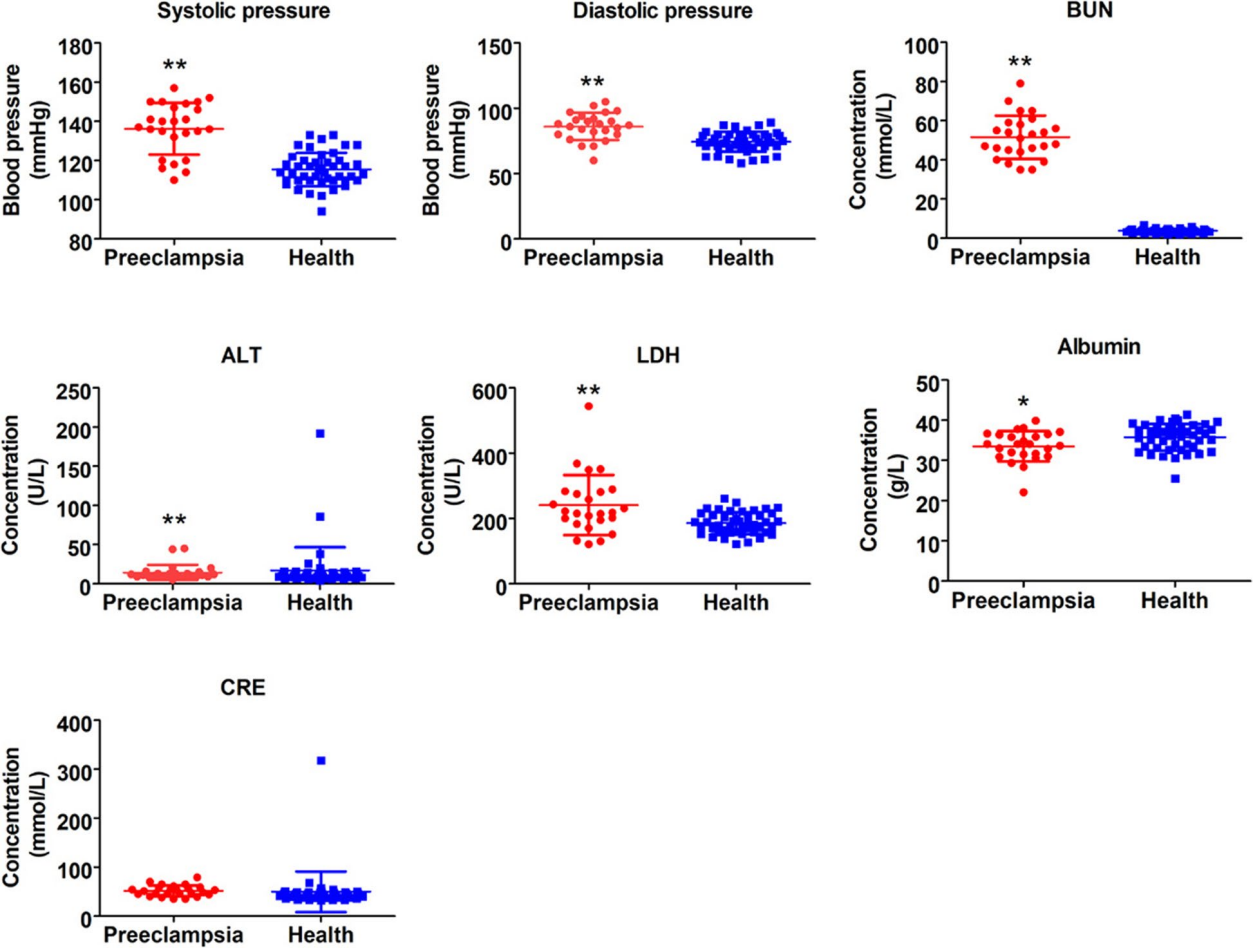
Routine clinical diagnostic index of preeclampsia. **P < 0.01 vs healthy controls; *P < 0.05 vs healthy controls; *n* = 40; *t*-test

**Fig. 6:**
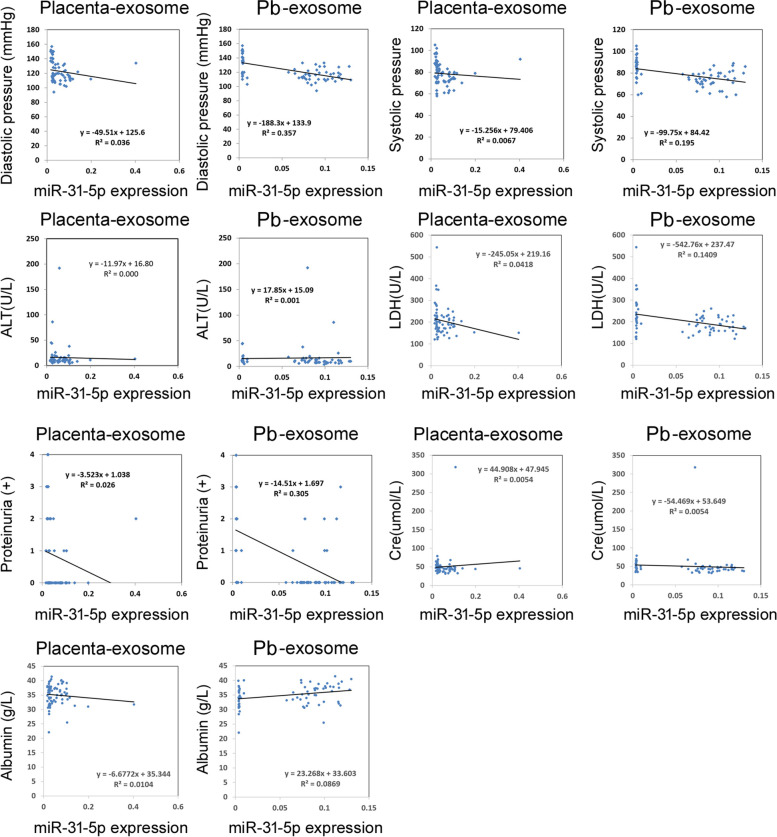
Correlation between exosome-derived miR-31-5p and routine clinical diagnostic indicators of preeclampsia. Pb, peripheral blood

To determine whether exosome-derived miR-31-5p is correlated with routine clinical diagnostic indicators of preeclampsia, we first analyzed differences in routine clinical diagnostic indicators between pregnant women with preeclampsia and healthy pregnant women. In pregnant women with preeclampsia, systolic and diastolic pressure, proteinuria, alanine aminotransferase, lactate dehydrogenase, blood urea nitrogen, and albumin levels were significantly higher than those in healthy pregnant women (Fig. 5). Correlation analysis indicated that the association between miR-31-5p and routine clinical diagnostic indicators of preeclampsia was basically consistent both in the placenta and peripheral blood. mir-31-5p was negatively correlated with systolic and diastolic pressure, lactate dehydrogenase, and proteinuria (Fig. 6).

## Discussion

The placenta is a chimera of maternal decidual cells and fetal trophoblasts. It is formed when fetal membranes become closely attached to the uterine wall, and the process involves interaction of the syncytial trophoblast, villi trophoblast, villi trophoblast and vascular trophoblasts belonging to the fetal part and the uterine myometrium, endometrial decidua, and spiral artery [1–4]. In preeclampsia, placental perfusion is reduced in early pregnancy when the invasion of extravillous trophoblasts is disturbed, resulting in reduced spiral artery remodeling. Extravillous trophoblasts are unable to sufficiently invade the decidua and myometrium, the spiral arteries remain narrow, and placental neovascularization is disrupted. Consequently, placentation is shallow, which leads to reduced placental perfusion. These effects result in increased production of humoral factors that injure or activate endothelial cells in the uteroplacental circulation. Eventually, these factors are introduced into the systemic circulation and affect many maternal organs [1–4]. Preeclampsia can prove to be fatal to pregnant women and fetus, so early detection and prompt treatment are pivotal. Routine clinical diagnostic indicators can prove to be helpful; however, the currently known markers of preeclampsia are relatively broad and demonstrate low specificity [1–4]. The relationship between the abnormal regulation of miRs and the occurrence and development of preeclampsia is being gradually revealed. In recent years, an increasing number of studies have reported that peripheral blood exosomes can serve as a potential, sensitive, noninvasive biomarker for disease diagnosis and evaluation.

Herein we first determined whether exosomes containing specific miRs exist in the placenta and peripheral blood of pregnant women with preeclampsia. Subsequently, we studied the *DLK1-Dio3* imprinted miR cluster. Our experimental results validated our hypothesis. Secretion of placental exosomes was found in pregnant women with preeclampsia; furthermore, the *DLK1-Dio3* imprinted miR cluster comprised 10 miRs, the expression levels of which were significantly different from those in healthy controls. Interestingly, the expression level of the *DLK1-Dio3* imprinted miR cluster was consistent between peripheral blood and placental exosomes of pregnant women with preeclampsia. Finally, we need to verify whether these miRs are correlated with routine clinical diagnostic indicators of pregnant women with preeclampsia, so as to confirm that exosome miRs are consistent with the above indicators.

In terms of exosome-derived miR-31-5p, we found that the expression trend of miR-31-5p was closely correlated with multiple routine diagnostic indicators of preeclampsia. These results suggest that miR-31-5p can serve as a potential biomarker for evaluating the progression of preeclampsia. miR-31-5p evidently regulates the TGF-β signaling pathway and affects epithelial–mesenchymal transition [32]. Abnormal expression of the TGF-β signaling pathway in trophoblasts can lead to the development of preeclampsia [33]. miR-31-5p reportedly also plays a vital role in angiogenesis. A study reported that atrial-specific upregulation of miR-31 in human atrial fibrillation is an important mechanism that causes atrial loss of dystrophin and neuronal nitric oxide synthase; this loss leads to the electrical phenotype induced by atrial fibrillation [34]. Neuronal nitric oxide synthase is evidently closely related to hypertension and preeclampsia [35, 36]. Based on previous studies, we believe that miR-31-5p has a profound impact on the emergence of the clinical manifestations of preeclampsia. Moreover, our findings indicate that miR-31-5p exists in the placenta and peripheral blood of pregnant women with preeclampsia, which further validates the relationship between miR-31-5p and preeclampsia-related symptoms.

## Conclusions

To summarize, we speculate that in pregnant women with preeclampsia, exosome-derived miR-31-5p can serve as an effective and sensitive biomarker to evaluate the progression of preeclampsia.

## Data Availability

The datasets used or analysed during the current study are available from the corresponding author on reasonable request.

## Abbreviations

miRs: microRNAs
DLK1-DIO3: Delta-like homolog 1 gene and the type III iodothyronine deiodinase
qPCR: Quantitative real-time PCR
H&E: Hematoxylin-eosin staining
TEM: Transmission electron microscopy
Pb: Peripheral blood
KEGG: Kyoto Encyclopedia of Genes and Genomes
